# Hallux valgus and plantar pressure loading: the Framingham foot study

**DOI:** 10.1186/1757-1146-6-42

**Published:** 2013-10-20

**Authors:** Andrew M Galica, Thomas J Hagedorn, Alyssa B Dufour, Jody L Riskowski, Howard J Hillstrom, Virginia A Casey, Marian T Hannan

**Affiliations:** 1 Institute for Aging Research at Hebrew Senior Life, 1200 Centre Street, Boston, MA, USA; 2 Beth Israel Deaconess Medical Center, Boston, MA, USA; 3 Harvard Medical School, Boston, MA, USA; 4 Glasgow Caledonian University, Glasgow, UK; 5 Hospital for Special Surgery, New York, NY, USA

## Abstract

**Background:**

Hallux valgus (HV), a common structural foot deformity, can cause foot pain and lead to limited mobility. The purpose of this study was to evaluate differences in plantar pressure and force during gait by HV status in a large population-based cohort of men and women.

**Methods:**

A trained examiner performed a validated physical examination on participants’ feet and recorded the presence of hallux valgus and other specific foot disorders. Each foot was classified into one of four mutually exclusive groups based on the foot examination. Foot groups were: (i) HV only, (ii) HV and at least one additional foot disorder (FD), (iii) no HV but at least one other FD, and (iv) neither HV nor FD (referent). Biomechanical data for both feet were collected using Tekscan Matscan. Foot posture during quiet standing, using modified arch index (MAI), and foot function during gait, using center of pressure excursion index (CPEI), were calculated per foot. Further, walking scans were masked into eight sub-regions using Novel Automask, and peak pressure and maximum force exerted in each region were calculated.

**Results:**

There were 3205 participants, contributing 6393 feet with complete foot exam data and valid biomechanical measurements. Participants with HV had lower hallucal loading and higher forces at lesser toes as well as higher MAI and lower CPEI values compared to the referent. Participants with HV and other FDs were also noted to have aberrant rearfoot forces and pressures.

**Conclusions:**

These results suggest that HV alters foot loading patterns and pressure profiles. Future work should investigate how these changes affect the risk of other foot and lower extremity ailments.

## Background

Hallux valgus (HV), a structural foot deformity often resulting in a reactive soft tissue bunion, can cause foot pain and limited mobility [1]. Women are twice as likely to have this condition [2,3] and older adults have a higher prevalence of HV (23% aged 18–25 years versus 35.7% over age 65 years) [4]. Footwear has also been implicated in the development of HV; especially shoes with higher heels or improper fit [5].

The degree to which foot anatomy or biomechanics influence HV is poorly understood. In a 2012 systematic review and meta analysis, Nix et al. reported that the first intermetatarsal angle and first metatarsal protrusions distance were significantly associated with hallux valgus, but also noted that a number of radiographic factors were not significantly associated with hallux valgus [6]. Arch height is noted as an area of interest in clinical models of hallux valgus [7], which often cite low arches as a contributing factor. However, past research has yielded inconsistent results [6]. While Nguyen et al. found a significant association between a clinical assessment of pes planus and hallux valgus in men [2], Kilmartin et al. reported no relation between arch height and hallux valgus when using an arch index [8]. Similarly, studies are inconsistent regarding whether a curved joint head was [9] or was not [10] associated with HV.

Studies that report on plantar pressure distributions for individuals with and without HV are also inconclusive. For example, loading at the hallux may be reduced [11-13], increased [14], or unaffected [15] by the presence of HV. These studies were limited by small sample sizes [14-16] and narrow age ranges [15,17], as well as exclusions of severe clinical cases [14], men [18] , and feet with multiple foot disorders [18].

Prior studies are limited by conflicting results, small sample sizes, and consideration of hallux valgus in isolation of concurrent foot disorders. Addressing these limitations can impact clinical decision making and evaluation of treatment strategies. The purpose of this study is to describe plantar pressures and forces in a large epidemiologic, population-based study of older adults and to investigate whether these measures differ between those with and without HV and other foot deformities. We hypothesized that the presence of HV is associated with decreased loading under the hallux and resultant offloading under the forefoot.

## Methods

### Study population

Study participants were members of the Framingham Foot Study [18], a population-based study comprised of three cohorts: Framingham Original Cohort, Framingham Offspring Cohort and a community sample drawn from the town of Framingham, MA, USA [18]. Briefly, the Framingham Original Cohort was derived in 1948 from a two-thirds population-based sample of the town of Framingham, MA, USA, while the Framingham Offspring cohort was composed of a sample of the adult children and spouses of the Original Cohort. Members of these cohorts between 2002–2008 were included in the Framingham Foot study along with a newly recruited community sample. The Framingham community sample was recruited via census-based, random digit-dial of ambulatory residents who were age 50 or older. The Framingham Foot Study was approved by the Institutional Review Boards at Hebrew SeniorLife and Boston University. All study participants provided written, informed consent prior to enrolment. Between 2002 and 2008, Foot Study participants received a physical and biomechanical assessment of their feet. For this analysis, only Framingham Foot Study participants with complete foot biomechanical data and foot disorder data were included.

### Hallux valgus and other foot disorders

A podiatric-trained examiner performed a validated physical examination on participants’ feet and recorded the presence of specific foot disorders including hallux valgus, hallux rigidus, claw toes, hammers toes, and overlapping toes [2]. The validity of the foot exam was previously evaluated in a sample of elderly residents by comparing podiatry clinic findings to the results from the trained study examiners. There was excellent agreement for HV as well as other foot disorders that were included in the foot examination. A comparison of multiple examiners yielded kappa values >0.85 (all *p* < 0.01), and all domains demonstrated excellent interobserver and intraobserver reliability [19,20]. Presence of HV (yes/no) was defined as a 15° or greater abduction of the hallux with respect to the first metatarsal. While the participant was standing, the examiner compared the angle of the hallux to an illustration of a 15° angle printed on a laminated page, and recorded hallux valgus as present if the angle was larger than the illustration. Hallux valgus, hammer toes, claw toes, and overlapping toes were assessed during weight-bearing stance. Hallux rigidus was measured while the participant was non-weight bearing and was considered present if the hallux was frozen or rigid during attempted passive movement by the examiner. All foot disorders (FD) were recorded as present or absent.

Each foot was classified into one of four mutually exclusive groups based on the physical examination. The foot groups were defined as: (i) hallux valgus only (HV-only), (ii) HV and at least one additional foot disorder (HV + FD), (iii) no HV but at least one other foot disorder (no HV-FD only), and (iv) no-HV and no-FD (referent group).

Age, sex and weight were also recorded at the time of examination. Weight was measured to the nearest half pound using a standardized balance beam scale and converted to Newtons.

### Plantar pressure data collection

Plantar pressure data were collected using a Tekscan Matscan (Tekscan Inc., Boston MA) pressure mat with a capture rate of 40 Hz, which was sufficient for the type of data collected and has moderate to good reliability [21]. A scan of each participant in quiet, bipedal stance was collected. Additionally, participants were instructed to walk at a self-selected pace across the mat. A single pressure scan of each foot was recorded using the two-step method [22], which entails the participants stepping on the pressure map on the second step. The two-step method has been shown to be as reliable as data collection using the mid-gait approach [22]. The time constraints associated with a large epidemiological study allowed recording of a single walking trial.

### Plantar pressure analysis and outcome measures

**Figure 1:**
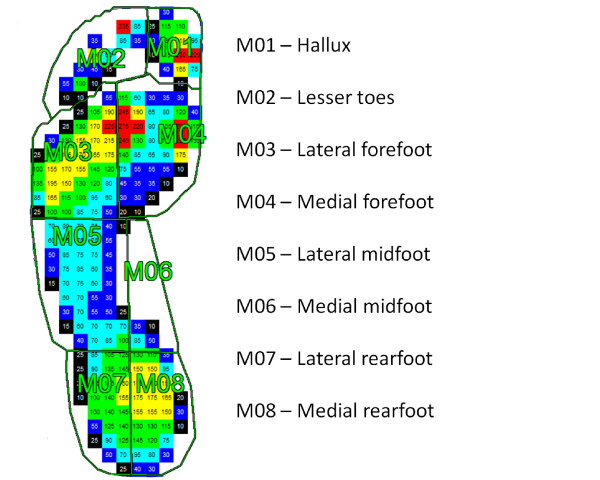
The eight region foot mask used to analyze Matscan plantar pressure scans in the Framingham Foot Study, 2002–2008.

Walking scans were masked into eight sub-regions using Novel Automask (Novel GmbH, Munich, Germany), and the peak pressure and maximum force exerted in each region were calculated. The eight regions were: (i) hallux, (ii) lesser toes, (iii) lateral forefoot, (iv) medial forefoot, (v) lateral midfoot, (vi) medial midfoot, (vii) lateral rearfoot, and (viii) medial rearfoot (Figure 1).

**Figure 2:**
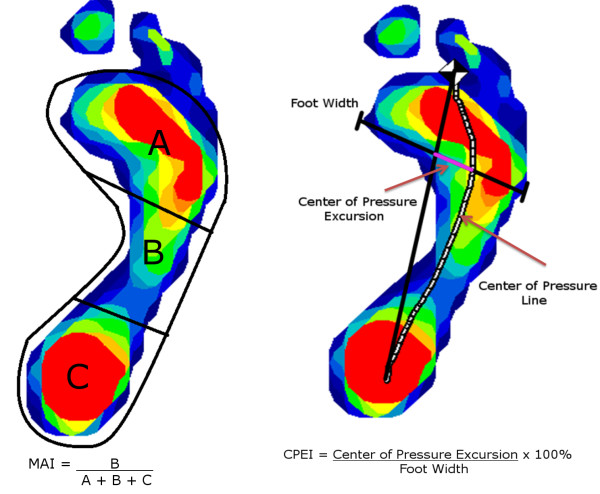
**Calculation of the center of pressure excursion index (CPI) and the modified arch index (MAI) in the Framingham Foot Study, 2002–2008.** Used with permission by Arthritis Care & Research; John Wiley & Sons, Inc.

In addition to regional pressures and force measurements, the center of pressure excursion index (CPEI) [23], a measure of foot function during gait [23], and the modified arch index (MAI), a measure of foot structure [24], were calculated for each foot. CPEI is related to clinical foot type and was defined as the excursion of the center of pressure from a constructed line connecting the first and last points of a center of pressure curve measured in the distal tertile of the foot and normalized by the foot’s width [23]. Lower CPEI values indicate a more pronated foot during gait, whereas higher CPEI values indicate more supination (Figure 2). MAI, calculated from the static weight-bearing stance scan was calculated by dividing each foot, not including the toes, lengthwise into three equal segments. The pressure under the middle third of the foot was divided by the pressure under the entire foot to yield the MAI [24]. Previous work has shown that MAI was inversely associated with navicular height, with higher MAI values indicating a lower arch [25].

### Statistical analysis

Means and standard deviations, or frequencies, where appropriate, were calculated for the overall population, and separately by foot group. A per-foot analysis using linear regression was used to determine the association between each biomechanical measure and foot group, both crude and adjusted (age, sex, and weight [Newtons]). Generalized Estimating Equations (GEE) were used to account for the correlation between right and left foot of the same person. The dependent variables were peak pressure and maximum force in each of the foot regions, along with CPEI and MAI. Results were considered statistically significant at *p* < 0.05. *P*-values from the linear regression models were adjusted for the 8 comparisons made within the mask in models of peak pressure and maximum force using a Bonferroni correction for multiple comparison testing. *P*-values from models of CPEI and MAI were not adjusted. The results presented account for the correction for multiple testing. All statistical analyses were conducted using the SAS statistical analysis package, version 9.3 (SAS Institute, Cary, NC).

## Results

### Population

**Figure 3:**
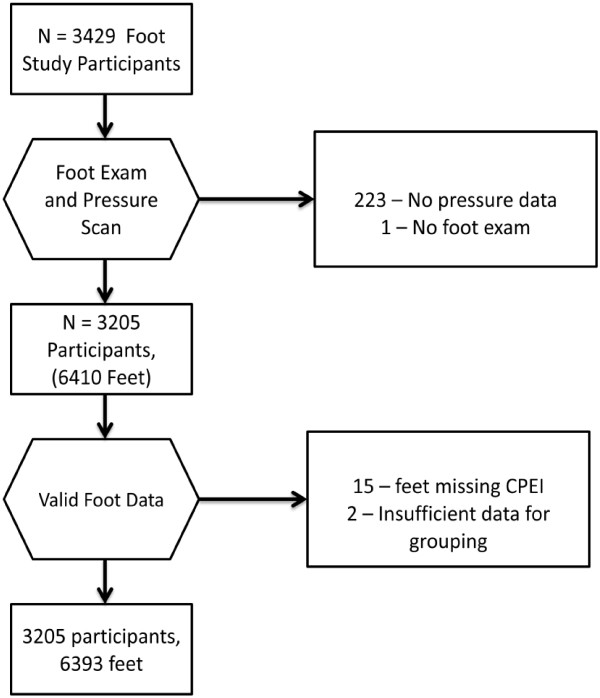
Flow of data for the participants included in the analysis of Framingham Foot Study data, 2002–2008.

There were 3205 participants, contributing 6393 feet with complete foot exam data and valid biomechanical measurements (Figure 3). The average age was 66 years and 56% of the sample was female (Table 1).

**Table 1:** Characteristics of the Framingham foot study population, 2002-2008*

	**Population**	**No Hallux Valgus/No foot disorder (referent)**	**Hallux Valgus only**	**Hallux Valgus + foot disorder**	**Foot disorder only**
N feet	6393	3707	1123	641	922
Age, years	66.2 ± 10.5	64.3 ± 9.7	65.2 ± 10.0	73.4 ± 10.9	69.9 ± 10.9
Female (n,%)	1799 (56.1%)	1849 (49.9%)	832 (74.1%)	462 (72.1%)	448 (48.6%)
Weight, pounds	174.1 ± 39.5	178.4 ± 39.5	164.4 ± 36.0	161.7 ± 38.9	177.3 ± 39.9
BMI, kg/m^2^	28.4 ± 5.5	28.8 ± 5.5	27.7 ± 5.2	27.5 ± 5.4	28.5 ± 5.3

*Data reported as mean ± standard deviation, unless otherwise noted.

### Maximum forces

Statistically significant differences in maximum force under the hallux were seen in both HV groups (i.e., HV-Only, HV + FD), but not in the No-HV + FD after adjustment for age, sex, and weight (Table 2). In addition to reduced loading under the hallux, the HV-only group had increased loading under the lesser toes in both crude and adjusted models. Though crude associations suggested decreased loading in additional regions, the HV-only group was not significantly different from the referent group at the other masked regions in adjusted models. By comparison, the HV + FD group had significantly reduced loading under both the lateral forefoot and lateral rearfoot in addition to the aforementioned reduction under the hallux in both crude and adjusted models. After adjustment, the HV + FD group did not differ significantly from the referent in any other regions. The No-HV + FD group did not have any statistically significant differences in force in the adjusted models relative to the referent group, though there were significant crude associations. No significant differences were seen for any of the foot groups at the lateral or medial midfoot.

**Table 2:** Distributions of foot biomechanical variables in each of the four foot groups, Framingham foot study, 2002–2008

**Variable**	**No Hallux Valgus / No foot disorder (referent)**	**Hallux Valgus only**	**Hallux Valgus + foot disorder**	**Foot disorder only**
Maximum Force (N)				
Hallux	78.4 (39.70)	64.9 (41.72)^†^*	47.7 (33.47)^†^*	70.4 (42.28)^†^
Lesser Toes	41.1 (27.12)	45.3 (38.04)*	40.7 (25.91)^†^	38.1 (27.83)
Lateral Forefoot	249.7 (102.50)	225.7 (104.30)^†^	206.2 (84.81)^†^*	229.8 (110.81)^†^
Medial Forefoot	132.9 (72.03)	123.1 (63.02)^†^	116.4 (62.89)^†^	130.3 (76.12)
Lateral Midfoot	116.5 (94.35)	108.3 (93.37)	109.5 (76.57)	124.4 (112.22)
Medial Midfoot	18.8 (24.62)	21.0 (32.87)	20.0 (24.17)	21.6 (27.84)
Lateral Rearfoot	175.6 (63.30)	163.9 (59.92)^†^	151.9 (51.41)^†^*	167.1 (70.77)^†^
Medial Rearfoot	196.5 (68.11)	181.8 (65.06)^†^	172.6 (56.38)^†^	191.9 (73.32)
Peak Pressure (kPa)				
Hallux	218.6 (102.01)	212.0 (106.44)^†^	171.3 (99.38)*	206.0 (106.66)^†^
Lesser Toes	138.8 (77.36)	144.5 (87.82)^†^	148.3 (71.93)*	152.2 (90.16)^†^*
Lateral Forefoot	251.3 (88.12)	240.3 (89.11)^†^	231.5 (71.15)^†^	249.6 (88.87)
Medial Forefoot	212.8 (101.25)	206.5 (99.94)	207.2 (86.07)	222.0 (104.89)^†^
Lateral Midfoot	108.8 (64.45)	106.3 (74.1)	107.8 (61.02)	113.1 (72.42)
Medial Midfoot	74.4 (53.20)	73.5 (65.18)	68.1 (39.27)	73.4 (55.17)
Lateral Rearfoot	216.8 (80.05)	208.2 (80.42)^†^	200.4 (62.83)*	213.1 (83.86)
Medial Rearfoot	230.0 (85.98)	222.3 (84.72)^†^	215.3 (67.21)*	233.1 (92.31)
Center of Pressure Excursion Index	14.7 (8.07)	13.0 (8.00)^†^*	12.5 (7.74)^†^*	14.5 (8.00)
Modified Arch Index	0.102 (0.0809)	0.098 (0.079)^†^	0.112 (0.095)*	0.116 (0.099)^†^*

Means (standard deviations).

CPEI = center of pressure excursion index.

†= significantly different from referent group in crude model at *p* < 0.05 with Bonferroni adjustment.

*= significantly different from referent group in adjusted model (age, weight, sex) at *p* < 0.05 with Bonferroni adjustment.

### Peak pressures

There were no significant differences in peak pressure observed in the HV-only group in crude or adjusted models, but several differences were noted in the HV + FD group. In this group, in both crude and adjusted models, peak pressure was significantly reduced under the hallux, increased under the lesser toes, and reduced in both the medial and lateral rearfoot. In the No-HV + FD group, a similar increase in pressure was observed under the lesser toes, but no changes were observed in any other masked region.

### Center of pressure excursion index (CPEI) and modified arch index (MAI)

CPEI was significantly reduced in both the HV-only and HV + FD groups, but was not statistically significantly different in the No-HV + FD group. MAI, by comparison, was not significantly different in the HV-only group, but it was higher than the referent in the HV + FD and the No-HV + FD groups. These associations were maintained in both crude and adjusted models.

## Discussion

The purpose of this study was to evaluate the differences in plantar pressure and force during gait by hallux valgus (HV) status in a large population-based cohort of men and women. Our results show that loading in the hallux region was lower in participants with HV-only and in those with HV and other foot disorders, compared to those who had neither. All groups (HV-only, HV + FD, and FD-only) had greater loading or pressure in the lesser toe regions when compared to the referent. The HV + FD group also had lower lateral rearfoot maximum force and lateral and medial peak pressures relative to the referent. Furthermore, feet in the HV + FD group were more likely to display a lower center of pressure excursion index (CPEI) values, higher modified arch index (MAI) values, and reduced lateral rearfoot force and lowered rearfoot peak pressures compared to the referent group. These results suggest that feet with HV have altered loading patterns and pressure profiles that may put them at greater risk of other foot and lower extremity ailments.

### Hallux and lesser toes

Our study showed reduced force under the hallux in both HV groups (i.e., HV-only and HV + FD), but not in the No-HV + FD group. Pressure was also reduced under the hallux in the HV + FD group. While reduced pressure under the hallux in those with hallux valgus has been seen in previous studies [13,17], this result has not been reported consistently [26]. Past studies have also observed no significant difference in loading of the hallux [15], as well as an increase in pressure under the hallux [14]. The conflicting findings noted by Martinez-Nova et al. [14] could be due to the inclusion of only mild cases of hallux valgus and a comparatively smaller, younger cohort (mean age 54.7 years). Biomechanical studies have suggested a number of mechanisms to explain the reduced loading in the hallux region in feet with HV, including first ray hypermobility. In this model the first metatarsal gives way, resulting in an offloading of the hallux onto other aspects of the foot such as the second metatarsal [9,16,17,27]. The offloading of the hallux may be due to the reduced ability of the hallux to bear load [27], or an adaptation to pain. Correspondingly, we found reduced loading at the hallux region with HV, while maximum force in the lesser toe area was increased in the HV-only group. As this study is cross-sectional is it unclear if the differences in halluca l loading associated with HV are a result of HV or causative of it.

We also noted higher peak pressure under the lesser toes in those feet with other structural foot disorders (i.e., HV + FD and No-HV + FD groups). As noted above, the offloading of the hallux may be due to its reduced ability to bear load [27], or as an adaptation to pain. In the HV + FD and No-HV + FD groups, the lack of significant changes in force at the lesser toes points to a different explanation. As the other foot disorders considered in this study are primarily structural disorders of the toes, it is possible that the contact area under the toes is reduced in these two groups as a result of other foot disorders, which can explain increases in pressure despite the lack of significant changes in force in those with the other structural foot disorders.

### Forefoot

Prior studies of surgical patients have also reported that HV is associated with lower loading at the 1^st^ metatarsophalangeal joint (MTPJ) [11,12]. This reduced loading has been theorized to result from an elevation of the 1^st^ MPJ during gait, which limits the load this area is able to accept in those with HV [16,17,28]. Studies in non-clinical groups by comparison have noted an increased load at the 1^st^ MTPJ [15,16,26]. In this analysis, there were no differences with adjusted models in the medial forefoot in any of the foot disorder groups. As the masking used in this study did not differentiate between the individual metatarsals, it possible that the offloading of the first metatarsal head was offset by increased force on the 2^nd^ MPJ. Nonetheless, no significant differences were seen in the medial forefoot, even in feet with HV. While no changes were observed in the medial forefoot, the HV + FD group had significantly reduced loading in the lateral half of the forefoot. Lower force in the lateral forefoot has not been previously attributed to HV. One explanation for the lower loading in the lateral forefoot could be a more pronated foot during gait, which is typically thought to be associated with HV. This hypothesis was supported by the significantly lower CPEI value observed in the groups with HV.

### Rearfoot

Of the studies that have reported on the midfoot and rearfoot regions, there was no subdivision of these areas into a medial and lateral section [16,29], and only one study [29] showed a difference in either the midfoot or rearfoot pressures between feet with and without HV. Nyska et al. noted that feet prior to undergoing corrective surgery for HV displayed lower rearfoot pressure relative to those without the foot disorder [29]. In our study, a lower maximum force in the lateral rearfoot and lower rearfoot peak pressure were observed in the HV + FD group. A similar association of a medial to lateral loading pattern in people with rheumatoid arthritis suggests the rearfoot and forefoot may be coupled and the rearfoot may play a role in forefoot complications [30]. The medial to lateral loading pattern arises from a valgus rearfoot alignment and leads to excessive stress at the subtalar joint and forefoot region [31]. Although rearfoot alignment was not evaluated in this current study, the lower values of the CPEI noted in the groups with HV suggest that they displayed a valgus rearfoot alignment [23]. In short, these results suggest that the rearfoot may be an important factor in the etiology and treatment of HV. This novel aspect of our findings would need further investigation of these patterns of results in future studies.

### Center of pressure excursion index (CPEI) and modified arch index (MAI)

Biomechanical modelling of HV has reported associations of pes planus foot type with the etiology of HV [28]. However, clinical and biomechanical studies of HV have not yielded consistent results to implicate a particular foot type with HV [7,32]. In this current study, a smaller CPEI was observed in both HV-only and HV + FD groups. This indicates that participants with HV have a more pronated foot during gait relative to the referent group and may be more likely to have a pes planus foot type, which has been associated with a lower CPEI [23]. This observation is further supported in the HV + FD group by higher MAI values, indicating a lower arch in this group relative to the referent. These results offer support that HV is associated with differences in foot structure and function. Further research is needed to expand these novel findings.

### Strengths and limitations

While this study provides important insights into the foot biomechanics associated with hallux valgus, there are several limitations. As the study design was cross-sectional, causal relations cannot be inferred. Nonetheless, associations between foot groups and loading offer an epidemiological view of common loading profiles in those with HV and foot disorders. Only a single walking trial for each foot was recorded, which may have increased measurement error. However, random error would only serve to obscure weaker relations in these data rather than create false positive associations [33]. Moreover, the large study sample greatly mitigates this issue, as it is more than sufficiently powered even with a single scan [34].

As a common mask was used to define foot regions for all pressure scans, it is possible that foot regions may not have correlated exactly with the anatomical location of the corresponding metatarsals in some cases. To more accurately define foot regions, it may be useful in future studies to align anatomical foot structure from spiral X-ray tomography with plantar pressure data as described by Hastings et al. [35].

Although studies have previously addressed the topic of plantar loading in HV, our study was unique in that it was population-based and included both men and women and individuals with additional foot disorders. Recent studies of plantar loading have been limited by relatively small samples (~ 300 participants) [14,16]. Thus, the current study with over 3000 participants with information on multiple foot disorders may offer insights into the associations of HV that smaller studies cannot. Our study which included participants with HV and additional structural foot disorders, a typical exclusion for other HV studies [14], provides an understanding of how foot function is influenced when HV is coupled with additional structural foot disorders.

## Conclusions

Although the pathogenesis of HV is complex, a better understanding of HV and its clinical outcomes can be achieved by evaluating the validity of theoretical, kinematic, and radiographic results through plantar pressure loading. Plantar loading can assess the functional impact of a structural deformity as seen during gait, and this study has confirmed several key results in a population-based sample of adult men and women with HV. Namely, a lower hallucal loading was seen in participants with HV, with greater loading at the toes. HV and foot disorders was also associated with altered rearfoot forces, which given prior evidence suggesting forefoot complications are associated with rearfoot disorders, suggests that the rearfoot should be considered in etiology and treatment of HV and forefoot complications. In addition, lower CPEI and higher MAI values were associated with HV, confirming results from studies that have described foot pronation and lower arch structure in feet with HV. Prospective studies are needed to elucidate of the etiology of HV and structural disorders in relation to plantar pressure loading. Moreover, longitudinal studies of HV and foot disorders can track plantar pressure and loading changes that develop over time.

## Competing interests

The authors have no competing interests to report.

## Authors’ contributions

AMG contributed to the analysis and interpretation of data and drafted the original manuscript. TJH contributed to the analysis and interpretation of data and drafted the original manuscript. ABD carried out the statistical analyses, contributed to the interpretation of data and the revision of the manuscript. JLR participated in the interpretation of data and the drafting and revision of the manuscript. HJH participated in the study conception and design and provided critical revision of the manuscript for intellectual content. VAC made substantial contributions to the drafting and revision of the manuscript. MTH conceived of the study, was responsible for the acquisition of data, contributed to the analysis and interpretation of data, and provided critical revision of the manuscript for intellectual content. All authors read and approved the final manuscript.

## Authors’ information

Andrew M Galica and Thomas J Hagedorn share first authorship on this manuscript.
